# Integrated strain- and process design enable production of 220 g L^−1^ itaconic acid with *Ustilago maydis*

**DOI:** 10.1186/s13068-019-1605-6

**Published:** 2019-11-06

**Authors:** Hamed Hosseinpour Tehrani, Johanna Becker, Isabel Bator, Katharina Saur, Svenja Meyer, Ana Catarina Rodrigues Lóia, Lars M. Blank, Nick Wierckx

**Affiliations:** 1 iAMB-Institute of Applied Microbiology, ABBt-Aachen Biology and Biotechnology, RWTH Aachen University, Worringerweg 1, 52074 Aachen, Germany; 2 Institute of Bio- and Geosciences IBG-1: Biotechnology, Forschungszentrum Jülich, Wilhelm-Johnen-Str., 52425 Jülich, Germany

**Keywords:** *Ustilago maydis*, Itaconic acid, Metabolic engineering, Morphological engineering, Biochemical engineering, In situ precipitation

## Abstract

**Background:**

Itaconic acid is an unsaturated, dicarboxylic acid which finds a wide range of applications in the polymer industry and as a building block for fuels, solvents and pharmaceuticals. Currently, *Aspergillus terreus* is used for industrial production, with titers above 100 g L^−1^ depending on the conditions. Besides *A. terreus*, *Ustilago maydis* is also a promising itaconic acid production host due to its yeast-like morphology. Recent strain engineering efforts significantly increased the yield, titer and rate of production.

**Results:**

In this study, itaconate production by *U. maydis* was further increased by integrated strain- and process engineering. Next-generation itaconate hyper-producing strains were generated using CRISPR/Cas9 and FLP/FRT genome editing tools for gene deletion, promoter replacement, and overexpression of genes. The handling and morphology of this engineered strain were improved by deletion of *fuz7*, which is part of a regulatory cascade that governs morphology and pathogenicity. These strain modifications enabled the development of an efficient fermentation process with in situ product crystallization with CaCO_3_. This integrated approach resulted in a maximum itaconate titer of 220 g L^−1^, with a total acid titer of 248 g L^−1^, which is a significant improvement compared to best published itaconate titers reached with *U. maydis* and with *A. terreus*.

**Conclusion:**

In this study, itaconic acid production could be enhanced significantly by morphological- and metabolic engineering in combination with process development, yielding the highest titer reported with any microorganism.

## Background

More than 300 potential bio-based building blocks were selected from the U.S. Department of Energy according to criteria such as estimated processing costs, estimated selling price, and the technical complexity, to determine the most important chemicals that can be produced from biomass. In the top selection, nine belong to the group of organic acids [72], underlining the importance of this class of chemicals. One of these compounds is the unsaturated dicarboxylate itaconic acid. It was first described in 1837 [4] and primary reports about microbial production with *Aspergillus itaconicus* date back to 1931 [42]. Due to its two functional groups, radical polymerization of the methylene group and/or esterification of the carboxylic acid with different co-monomers is possible [59, 63, 67]. This leads to a wide range of applications in the paper, architectural, pharmaceutical, paint, lacquer, and medical industries [5, 6, 40, 43, 50, 55, 61, 71]. It can also be used as an intermediate for the production of 3-methyltetrahydrofuran, a potential biofuel with advantageous combustion properties [16]. Further, itaconate production by mammalian macrophages is reported, where it plays a key role in the human immune response [11, 57, 69], with possible applications as therapeutic agent for autoimmune diseases [2].

In spite of this wide variety of potential applications, the market size of itaconic acid in 2011 was relatively small, with 41,400 tons and a market value of $74.5 million [71]. This is caused by the relatively high price of approximately two dollars per kg and the availability of cheaper petro-based alternatives such as acrylic acid. Reduction of this price is, therefore, a major criterion for access to further markets. To be competitive against petro-based products, costs need to reduce to around $0.5 per kg [1]. Assuming that the price would decrease, itaconic acid has the possibility to replace acrylic acid in the production of poly(methyl methacrylate), the production of which is petroleum based with a market worth of $11 billion [39, 43, 59]. Since 1950, *Aspergillus terreus* is used for the industrial production of itaconate [59]. Charles Pfizer Co. was granted the first patent for the production of itaconate with the filamentous fungus *A. terreus* by submerged cultivation [37]. During the last decades, the responsible metabolic pathways and regulatory mechanisms of itaconate production in *A. terreus* were studied in detail [67]. Major advances were achieved through process development. This long history of optimization has enabled titers above 100 g L^−1^ and yields near the theoretical maximum at low pH, making *A. terreus* the current best production host for itaconate production [7, 29, 34, 49, 50, 53, 66]. However, despite the long history and experience, itaconate production in *A. terreus* remains challenging. A specific pellet growth form is required for high productivity [25, 39] and therefore, morphology has to be strictly controlled. *A. terreus* reacts very sensitively to certain medium impurities, which can induce mycelium formation and stop itaconate production [15, 48, 50]. Thus, medium must be pretreated to remove impurities from production medium, especially when using less pure industrial substrates such as molasses [38, 59]. Consequently, morphological control influences the manufacturing process tremendously, leading to increased operational costs and failed batches.

Besides *A. terreus*, numerous itaconate producers have been engineered in recent years, such as *E. coli* [26], *A. niger* [31], and *C. glutamicum* [60]. Besides these heterologous hosts, Ustilaginaceae like the pH-tolerant *Ustilago cynodontis* or the yeast-like *Ustilago maydis* are natural itaconate producers which have recently been engineered to higher efficiency [21, 24, 33, 75]. Among the Ustilaginaceae, *U. maydis* is the most studied species in the fields of plant pathogenicity, cell biology, and biotechnology [17, 18, 52, 68, 70]. The Ustilaginaceae produce a broad spectrum of interesting products such as organic acids [21, 24, 76], glycolipids [14, 58], polyols [21, 35], and enzymes [14]. This, along with their yeast-like growth, makes them attractive for biotechnological applications [21].

That said, certain stresses can induce filamentous growth in *U. maydis* [45, 54] but efficient itaconate production with this species is, at least at small scale, not coupled to a specific morphology. In wild-type *U. maydis*, itaconate production is induced by nitrogen limitation [74] and requires pH values above five [21]. Like in *A. terreus*, the genes encoding the itaconate production pathway in *U. maydis* are clustered and co-regulated [19, 53]. Considerable progress has been made in increasing the yield, titer, and rate of itaconate production in *U. maydis* and related species by metabolic engineering and process development. Geiser et al. [19] characterized the itaconate production pathway and identified an itaconate oxidase Cyp3, which produces the downstream product (*S*)-2-hydroxyparaconate. The disruption of this oxidase, and overexpression of the cluster-associated regulator Ria1, led to 4.5-fold increase in ITA production in *U. maydis* [18]. In *U. vetiveriae,* itaconate production from glycerol could be increased 2.5-fold by overexpression of *ria1* or 1.5-fold by overexpression of the mitochondrial transporter *mtt1* [75].

In another study, we could show that heterologous expression of the mitochondrial transporter MttA from *A. terreus* in *U. maydis* enables more efficient itaconate production than the native mitochondrial transporter [32]. Further, by deletion of *fuz7* in *U. cynodontis,* a stable yeast-like growth could be established for several relevant itaconic acid production conditions [33]. This is especially favorable for large-scale fermentation [62]. Furthermore, with optimization of growth media and the fermentation process, such as pulsed fed-batch strategies, product titers can be significantly increased [20, 21]. This is especially effective when combined with in situ product removal approaches such as reactive extraction or calcium precipitation [23, 43, 46, 75].

These optimizations have individually made a significant impact on the efficiency of itaconate production in *Ustilago*. In this study, we consolidate several of these metabolic and bioprocess engineering strategies to achieve itaconate titers that surpass those currently achieved by any other host.

## Results and discussion

### Engineering of a marker-free *U. maydis* MB215 for enhanced itaconate production

**Fig. 1:**
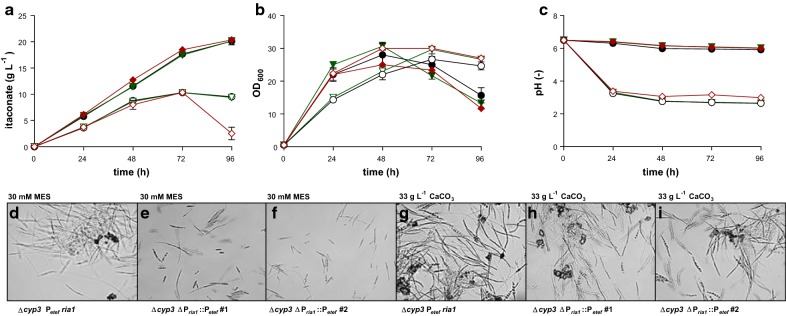
Itaconate production and growth of engineered *U. maydis* strains. Itaconate concentration (**a**), growth (OD_600_, **b**), pH (**c**) and DIC images at an magnification of 630× (**d**–**i**) with ∆*cyp3* ∆P_*ria1*_::P_*etef*_ #1 (green, filled inverted triangle) and ∆*cyp3* ∆P_*ria1*_::P_*etef*_ #2 (black, filled circle) in comparison to ∆*cyp3* P_*etef*_*ria1* (red, filled diamond) during shake flask cultivation in screening medium with 30 mM MES (open symbols) or 33 g L^−1^ CaCO_3_ (filled symbols) with 50 g L^−1^ glucose. Error bars indicate the standard error of the mean (*n* = 3)

Previously, Geiser et al. [18] reached titers up to 63.2 ± 0.7 g L^−1^ with production rates up to 0.38 ± 0.00 g L^−1^ h^−1^ and a yield up to 0.48 ± 0.02 g_ITA_ g_GLC_^−1^ in bioreactor experiments by deletion of the itaconate oxidase encoding *cyp3* gene and overexpression of the gene encoding transcriptional regulator Ria1 (∆*cyp3* P_*etef*_ *ria1*). Unfortunately, two out of five possible antibiotic resistance markers available for *U. maydis* were genomically incorporated in this design, which limited further modification steps. Recently, Schuster et al. [65] established a CRISPR/Cas9 system for *U. maydis* enabling scarless and marker-free genome editing [12]. This technology, along with the FLP/FRT system for marker recycling already used in *U. maydis* [41] removes previous limitations of available antibiotic markers. We re-engineered the modifications described by Geiser et al. [18] using the CRISPR/Cas9 system from Schuster et al. [65]. To delete *cyp3* and thereby abolis production of (*S*)-2-hydroxyparaconate, a repair template was used to delete the whole gene. It consisted of 1000 bp flanks homologous to sequences up- and downstream of *cyp3*. The overexpression of *ria1* was not achieved by the *in trans* insertion of an expression cassette at the *cbx* locus, but rather by a direct *in cis *replacement of the native P_*ria1*_ promoter by the strong and constitutive P_*etef*_ promoter. Here, the same strategy was chosen as for *cyp3*, including P_*etef*_ between the flanks of the repair templates. Promoter exchanges were previously shown to effectively upregulate native genes [20]. Two chosen transformants of the resulting strain (∆*cyp3* ∆P_*ria1*_::P_*etef*_ #1 and ∆*cyp3* ∆P_*ria1*_::P_*etef*_ #2) were compared to the control strain from Geiser et al. [18] in System Duetz^®^ 24-well plates [13], in screening medium with 50 g L^−1^ glucose, buffered either with 30 mM MES or 33 g L^−1^ CaCO_3_ (Fig. 1). As expected, itaconate production was lower using 30 mM MES compared to 33 g L^−1^ CaCO_3_, since *U. maydis* prefers pH values above 5 [21]. In both tested conditions, the transformants showed no difference to the control except for one notable exception. Itaconate concentrations in the cultures with the ∆*cyp3* P_*etef*_*ria1* strain decreased markedly at 96 h with 30 mM MES (Fig. 1a). This rapid decrease shows that *U. maydis* can degrade itaconate, likely through a similar pathway as that described for *A. terreus* [10, 18]. Possibly, the expression of the genes encoding this degradation pathway is affected by the promoter replacement which removed the native *P*_*ria1*_ promoter. For further investigations, we selected the strain *U. maydis* ∆*cyp3* ∆P_*ria1*_::P_*etef*_ #2.

In the cultures of these overproducing strains, we also observed a degree of filamentous growth. Although this is by far not as prominent as described for *U. cynodontis* [33], elongated cells and filaments were formed in all tested *U. maydis* strains for all conditions shown in Fig. 1, especially upon addition of CaCO_3_.

### Morphological engineering in *U. maydis* ∆*cyp3* ∆P_*ria1*_::P_*etef*_

Usually, filamentous growth in *U. maydis* is investigated in terms of pathogenicity. In its natural habitat, filamentous growth is indispensable to *U. maydis* for infection of *Zea mays*. This is strongly coupled with sexual development including a complex regulatory system [36, 51, 52]. Filamentous growth can also occur in haploid cells when they encounter stresses such as low pH, nitrogen limitation, or the presence of sunflower oil [45, 56]. This ability to grow filamentously is an obstacle in a biotechnological context, as it strongly influences bioprocess parameters such as oxygen transfer, viscosity, and clogging, and it increases the sensitivity to hydro-mechanical stress [44]. To solve this problem and to restore robust yeast-like growth, the f*uz7* gene was deleted in the marker-free ∆*cyp3* ∆P_*ria1*_::P_*etef*_ #2 strain by replacement with a hygromycin marker through homologous recombination, followed by FLP/FRT-mediated marker excision [41]. Fuz7 is part of the Ras/mitogen-activated protein kinase (MAPK) pathway, which plays an important role in conjugation tube formation and filamentous growth [3]. By deletion of *fuz7* in the strongly filamentous *U. cynodontis,* filamentous growth was repressed without influencing itaconate production and cell fitness under biotechnologically relevant conditions [33]. Deletion of *fuz7* in *U. maydis* is known to abolish filamentous growth, and it also renders the strain completely apathogenic [3, 45]. This inability to colonize the maize plant is an additional advantage in a biotechnological context, as it may alleviate possible regulatory hurdles for industrial application.

**Fig. 2:**
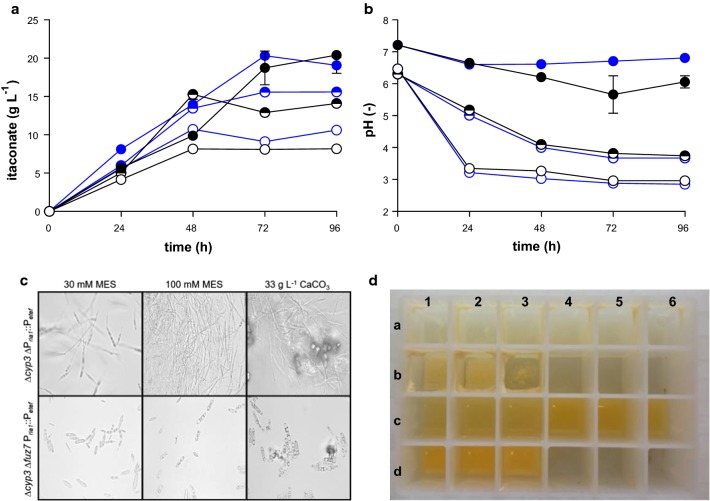
Itaconate production and growth of morphology-engineered *U. maydis* strains. *U. maydis* ∆*cyp3* ∆P_*ria1*_::P_*etef*_ (black, **d**_a1–6, b1–3_) and *U. maydis* ∆*cyp3* ∆P_*ria1*_::P_*etef*_ ∆*fuz7* (blue, **d**_c1–6, d1–3_). Itaconate concentration (**a**), pH course (**b**), DIC images at a magnification of 630 (**c**) and a 24 well plate during System Duetz^®^ cultivation in screening medium with 30 mM MES (open circle, **d**_a1–3+c1–3_), 100 mM MES (circle with upper half filled, **d**_a4–6+c4–6_) and 33 g L^−1^ CaCO_3_ (filled circle, **d**_b1–3+d1–3_) and 50 g L^−1^ glucose. Error bars indicate the standard error of the mean (*n* = 3)

To assess the effect of *fuz7* deletion, cultivation studies in screening medium with 30 mM and 100 mM MES, and 33 g L^−1^ CaCO_3_, were performed (Fig. 2). As expected, *U. maydis* ∆*cyp3* ∆P_*ria1*_::P_*etef*_ ∆*fuz7* grew completely yeast-like in all tested conditions. In contrast, *U. maydis* ∆*cyp3* ∆P_*ria1*_::P_*etef*_ grew filamentously (Fig. 2c), resulting in extensive adherence to the walls of the culture plates (Fig. 2d). This striking difference in morphology in the *fuz7* mutant greatly improves handling of these cultures, while it did not negatively affecting itaconate production. Rather, production was significantly better at the end of cultivation for 30 mM and 100 mM MES.

### Mitochondrial transporter engineering in *U. maydis* ∆*cyp3* ∆P_*ria1*_::P_*etef*_ ∆*fuz7*

**Fig. 3:**
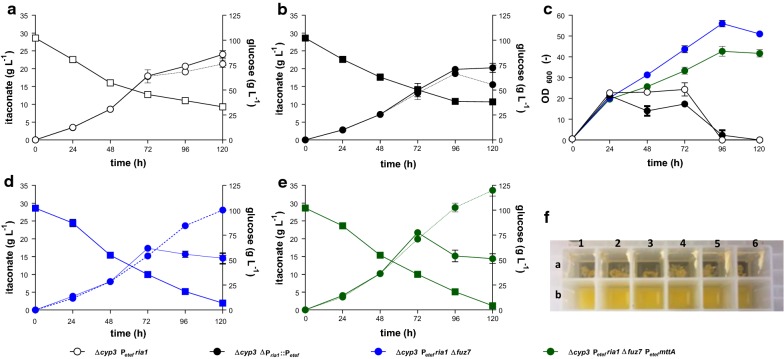
Comparison of aqueous and total itaconate concentrations in cultures of engineered *U. maydis* strains. Itaconate production (filled circle), glucose consumption (filled square) and macroscopic image of a 24-well plate after 96 h (**f**) of *U. maydis* ∆*cyp3* P_*etef*_*ria1* (**a**, **f**_(a1–3)_), ∆*cyp3* ∆P_*ria1*_::P_*etef*_ (**b**, **f**_(a4–6)_), ∆*cyp3* ∆P_*ria1*_::P_*etef*_ *∆fuz7* (**c**, **f**_(b1–3)_) and ∆*cyp3* ∆P_*ria1*_::P_*etef*_ *∆fuz7* P_*etef*_*mttA* (**d**, **f**_(b4–6)_) during System Duetz^®^ cultivation in screening medium with 66 g L^−1^ CaCO_3_ and 100 g L^−1^ glucose. Dotted lines represent samples treated with HCl and continuous lines represents untreated supernatant samples. Error bars indicate the standard error of the mean (*n* = 3). Glucose values were combined for untreated and treated samples (*n* = 6)

Recently, we could show by complementation experiments that overexpression of the mitochondrial transporter encoded by *mttA* from *A. terreus* enables higher itaconate production in *U. maydis* than overexpressing the native *mtt1* [32]. Thus, to further increase itaconate production, we expressed *mttA* of *A. terreus* in *U. maydis* ∆*cyp3* ∆P_*ria1*_::P_*etef.*_ ∆*fuz7* using plasmid pETEF_CbxR*_At_mttA* [32]. The best of three individual transformants was selected for further study. Upon cultivation of this transformant with CaCO_3_, a white precipitate was observed in samples of these cultures, indicating that the solubility limit of calcium itaconate was reached. As described for malic acid production with *U. trichophora*, and itaconate production with *U. vetiveriae*, calcium salts of these organic acids have a lower solubility, leading to in situ precipitation in cultures where high titers are reached, usually preceded by a transient supersaturation of the product [75, 77]. To assess the effect of in situ itaconate precipitation in the engineered *U. maydis* strains, they were cultivated in System Duetz^®^ plates in screening medium with 100 g L^−1^ glucose and 66 g L^−1^ CaCO_3_. Samples were analyzed with and without HCl treatment to re-solubilize the precipitated Ca-itaconate (Fig. 3). With the higher glucose concentration, the difference between strains with and without *fuz7* deletion becomes more apparent, with the filamentous strains having a lower substrate uptake rate and a residual glucose concentration between 33.1 ± 2.6 and 38.2 ± 2.9 g L^−1^. Consequently, the strain with *fuz7* deletion reached higher final titers, with *U. maydis* ∆*cyp3* ∆P_*ria1*_::P_*etef*_ ∆*fuz7* P_*etef*_*mttA* producing 33.6 ± 1.6 L^−1^ itaconate, which is 1.2-fold more than its predecessor ∆*cyp3* ∆P_*ria1*_::P_*etef*_ ∆*fuz7*. This experiment indicates a solubility of Ca-itaconate of 14.5 ± 0.6 g L^−1^ under the tested conditions, estimated from the endpoint aqueous concentrations of cultures shown in Fig. 3d, e. This value is below the measured itaconate concentrations of some samples from the abovementioned cultures (Fig. 2), indicating that these samples were in the supersaturation state and that some product may have been overseen due to precipitation.

An even more pronounced effect was observed with similar cultures using glycerol as C-source (Additional file 1: Fig. S1). Glycerol is a very poor substrate for wild-type *U. maydis* MB215 [77], and it invokes a high degree of filamentation and pigmentation in *U. cynodontis* [33]. The *fuz7* deletion had a very positive effect on the glycerol uptake rate and itaconate production, with the ∆*cyp3* ∆P_*ria1*_::P_*etef*_ ∆*fuz7* strain producing 13.1 ± 0.04 g L^−1^, compared to 4.3 ± 0.4 g L^−1^ produced by the ∆*cyp3* ∆P_*ria1*_::P_*etef*_ control strain. Titers could be further increased with *U. maydis* ∆*cyp3* ∆P_*ria1*_::P_*etef*_ ∆*fuz7* P_*etef*_*mttA* to 16.1 ± 0.4 g L^−1^ itaconate.

### Optimized itaconate production in a stirred bioreactor

**Fig. 4:**
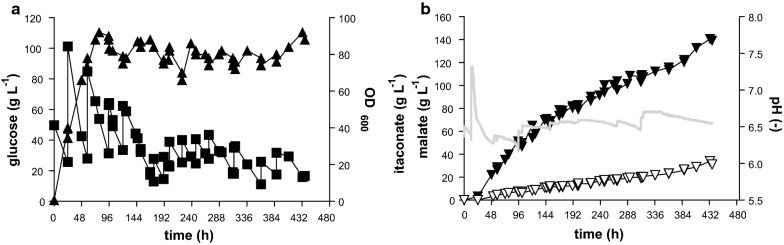
Controlled pulsed fed-batch fermentation of *U. maydis* MB215 ∆*cyp3* ∆P_*ria1*_::P_*etef*_ ∆*fuz7* P_*etef*_*mttA* in the presence of CaCO_3_. **a** Growth (filled triangle), and glucose concentration (filled square) and **b**: concentration of itaconate (filled inverted triangle), malate (open inverted triangle), and pH (continuous line) during a single representative bioreactor cultivation in batch medium with a starting concentration of 50 g L^−1^ glucose and 1.6 g L^−1^ NH_4_Cl. pH was kept above 6.2 by manual addition of CaCO_3_. Feeding schemes of glucose and CaCO_3_ are listed in Additional file 1: Table S1

In principle, the alleviation of product inhibition provided by the in situ precipitation of calcium itaconate enables much more extended cultures. In such cultures, productivity is only limited by the availability of the substrate and the stability of the biocatalyst. Therefore, to achieve high itaconate production, *U. maydis* MB215 ∆*cyp3* ∆P_*ria1*_::P_*etef*_ ∆*fuz7* P_*etef*_*mttA* was cultivated in pulsed fed-batch fermentations with CaCO_3_ in controlled 2-L bioreactors. The batch phase was started in screening medium containing 50 g L^−1^ glucose and 1.6 g L^−1^ NH_4_Cl (Fig. 4). The CaCO_3_ was added manually whenever pH dropped below 6.2, in the first 313 h as liquid suspension and after 313.5 h as a powder. Glucose was also pulsed into the fermenter to keep the concentration above 20 g L^−1^. The feeding schedule of CaCO_3_ and glucose is given in Additional file 1: Table S1. The resulting titer of 140 g L^−1^ itaconate was reached after 437 h. This is 2.2-fold more than the best published 63.2 ± 0.7 g L^−1^ from Geiser et al. [18] with *U. maydis*. Biomass formation mainly occurred in the first 72 h and reached OD_600_ values around 90, varying between 80 and 110 for the rest of the fermentation. These variations are likely due to analytical errors caused by CaCO_3_ precipitation upon sampling and OD measurement. An overall yield of 0.39 g_ITA_ g_GLC_^−1^ was reached and the overall productivity was 0.32 g L^−1^ h^−1^, with a maximum productivity between 24 and 120 h of 0.65 g L^−1^ h^−1^, after which it stayed relatively linear at 0.23 g L^−1^ h^−1^. This decrease in productivity might be caused by the high solids load of 10–15% CaCO_3_ and Ca-itaconate in the fermentation broth, which could result in inhomogeneous mixing with pockets of low oxygen tension. For itaconate production, sufficient supply of oxygen is very important, with even transient oxygen limitations leading to a decrease of production [27, 44]. Future process development should, thus, focus on better mixing with these high solids loads, i.e., by changing stirrer geometry which can promote better oxygen distribution in viscous media [9]. In addition to itaconate, production of 31 g L^−1^ malate was also observed thereby increasing the total acid production to 170 g L^−1^ and the total acid yield to 0.48 g_ACID_ g_GLC_^−1^. This increased by-product formation could be the result of the additional supply of CO_2_ by CaCO_3_. The efficient microbial production of malate via pyruvate relies on CO_2_ as co-substrate [77], and the additional CO_2_ provided by the CaCO_3_ might imbalance the precursor supply of itaconate.

In a similar approach where pH was controlled by titration with NaOH, a much lower level of itaconate production was observed (Additional file 1: Fig. S2), reaching a maximum titer of only 35.9 ± 1.5 g L^−1^ with a yield of 0.2 ± 0.01 g_ITA_ g_GLC_^−1^ and an overall productivity of 0.12 ± 0.004 g L^−1^ h^−1^. In this titrated fermenter less than 1 g L^−1^ malate was produced, supporting the hypothesis that the additional CO_2_ from CaCO_3_ increases malate production. The overall decrease of productivity in the titrated culture is likely caused by the overexpression of *mttA*, which significantly stresses the cells leading to reduced growth and productivity as described previously [32]. The application of in situ itaconate crystallization with CaCO_3_, greatly reduced product inhibition, which is especially relevant with this deeply engineered strain, leading to almost threefold higher production rates.

**Fig. 5:**
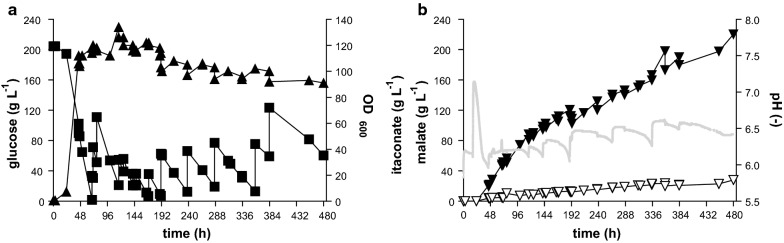
Controlled high-density pulsed fed-batch fermentation of *U. maydis* MB215 ∆*cyp3* ∆P_*ria1*_::P_*etef*_ ∆*fuz7* P_*etef*_*mttA* in the presence of CaCO_3_. **a** Growth (filled triangle), and glucose concentration (filled square) and **b** concentration of itaconate (filled inverted triangle), malate (open inverted triangle), and pH (continuous line) during a single representative bioreactor cultivation in batch medium with a starting concentration of 200 g L^−1^ glucose and 4 g L^−1^ NH_4_Cl. The pH was kept above 6.2 by manual addition of CaCO_3_. Feeding schemes of glucose and CaCO_3_ are listed in Additional file 1: Table S2

To further improve the production rate, the cell density was increased by increasing the NH_4_Cl concentration to 4 g L^−1^ and the starting glucose concentration to 200 g L^−1^ (Fig. 5). A similar feeding strategy of glucose and CaCO_3_ as above was applied (Additional file 1: Table S2). As expected, higher biomass formation was observed with the higher ammonium concentration, although the 2.5-fold higher nitrogen concentration only led to a moderate increase of the OD_600_ to around 110. A similar trend was observed with *A. terreus*, where a fourfold increase in phosphate as the growth-limiting nutrient only led to a twofold increase in biomass [48]. In spite of this, the overall production rate was increased significantly to 0.45 g L^−1^ h^−1^. The higher overall production rate was also reflected in a higher maximum rate of 0.74 g L^−1^ h^−1^ between 24 and 189 h followed by a fairly linear rate of 0.35 g L^−1^ h^−1^ for the rest of the fermentation. This higher rate enabled the production of 220 g L^−1^ itaconate and 28 g L^−1^ malate resulting in a total acid titer of 248 g L^−1^ in the same timeframe as the lower density culture. Of this total titer, approximately 14 g L^−1^ of each acid will be dissolved in the aqueous phase [77], with a further 287 g L^−1^ occurring as solid calcium salts. Occasional spikes in the measured itaconate concentration can be observed, likely due to the re-dispersion of Ca-itaconate clumps from the headspace into the broth. Indeed, extensive clumping could be observed owing to the very high solid loads of 20–35%. As expected, the higher rates come at a cost of a yield reduction to 0.33 g_ITA_ g_GLC_^−1^ and a total acid yield of 0.37 g_ACID_ g_GLC_^−1^, as more glucose is consumed for biomass production and maintenance.

## Conclusion

In this study, the combination of metabolic and morphological engineering together with in situ crystallization of itaconate yielded a titer of 220 g L^−1^ itaconate, which corresponds to 284 g L^−1^ calcium itaconate. This titer exceeds the 160 g L^−1^ achieved with *A. terreus* [48], although the yield and production rate achieved with *A. terreus* are still higher [50]. Especially, the yield achieved with *U. maydis* could be further increased by the reduction of byproduct formation, as illustrated by the relatively high levels of malate production under these conditions. The strategy of in situ crystallization has not been reported in a biotechnological context with *A. terreus*, likely because the used pH values and the presence of solids strongly affect its morphology [48]. The use of in situ crystallization greatly enhanced itaconate production, but it will also pose new bioprocessing challenges such as solid/solid separation of biomass, CaCO_3_ and Ca-itaconate, or pH shifts for resolubilization of itaconate prior to purification [47, 62]. In all, this study demonstrates the power of an integrated approach of strain and process engineering by greatly enhancing *Ustilago*-based itaconate production.

## Materials and methods

### Media and culture conditions

All strains used in this thesis are listed in Table 1. *E. coli* strains were grown in medium containing 10 g L^−1^ peptone, 5 g L^−1^ sodium chloride, 5 g L^−1^ yeast extract, and 5 g L^−1^ glucose. *U. maydis* strains were grown in YEPS medium containing, 10 g L^−1^ yeast extract, 10 g L^−1^ peptone, and 10 g L^−1^ sucrose. Growth and production experiments were performed using screening medium according to Geiser et al. [21] with varying glucose concentrations, C-sources (glycerol/glucose), and various buffer concentrations of 2-(*N*-morpholino) ethanesulfonic acid (MES) and 33 g L^−1^ CaCO_3_. This medium further contained 0.8 g L^−1^ NH_4_Cl, 0.2 g L^−1^ MgSO_4_·7H_2_O, 0.01 g L^−1^ FeSO_4_·7H_2_O, 0.5 g L^−1^ KH_2_PO_4_, 1 mL L^−1^ vitamin solution, and 1 mL L^−1^ trace element solution. The vitamin solution contained (per liter) 0.05 g d-biotin, 1 g d-calcium pantothenate, 1 g nicotinic acid, 25 g myo-inositol, 1 g thiamine hydrochloride, 1 g pyridoxol hydrochloride, and 0.2 g para-aminobenzoic acid. The trace element solution contained (per liter) 15 g EDTA, 0.45 g of ZnSO_4_·7H_2_O, 0.10 g of MnCl_2_·4H_2_O, 0.03 g of CoCl_2_·6H_2_O, 0.03 g of CuSO_4_·5H_2_O, 0.04 g of Na_2_MoO_4_·2H_2_O, 0.45 g of CaCl_2_·2H_2_O, 0.3 g of FeSO_4_·7H_2_O, 0.10 g of H_3_BO_3_ and 0.01 g of KI. Shaking cultures of *U. maydis* and mutants strains were performed in System Duetz^®^ (24 well plates) with a filling volume of 1.5 mL (*d* = 50 mm, *n* = 300 rpm, *T* = 30 °C and *Φ* = 80%) or in 500 mL shaking flasks with a filling volume of 50 mL (*d* = 25 mm, *n* = 200 rpm, *T* = 30 °C and *Φ* = 80%) [13]. If System Duetz^®^ was used, cultures were parallelly inoculated into multiple plates and for each sample point, and a complete plate was taken as sacrificial sample to ensure continuous oxygenation.

**Table 1:** *U. maydis* MB215 strains used in this study

Strain designation	Resistance	References
*Ustilago maydis* MB215		[30]
*Ustilago maydis* ∆*cyp3* P_*etef*_*ria1*	hyg^R^, cbx^R^	[18]
*Ustilago maydis* ∆*cyp3*		This study
*Ustilago maydis* ∆*cyp3* ∆P_*ria1*_::P_*etef*_ #1		This study
*Ustilago maydis* ∆*cyp3* ∆P_*ria1*_::P_*etef*_ #2		This study
*Ustilago maydis* ∆*cyp3* ∆*fuz7* ∆P_*ria1*_::P_*etef*_	hyg^R^	This study
*Ustilago maydis* ∆*cyp3* ∆P_*ria1*_::P_*etef*_ ∆*fuz7* P_*etef*_*mttA*	hyg^R^, cbx^R^	This study

Controlled batch cultivations were performed in a New Brunswick BioFlo^®^ 115 bioreactor (Eppendorf, Germany) with a total volume of 1.3 L and a working volume of 0.5 L or a total volume of 2.0 L and a starting volume of 1.0 L if CaCO_3_ was used. All cultivations were performed in batch medium containing 0.2 g L^−1^ MgSO_4_·7H_2_O, 0.01 g L^−1^ FeSO_4_·7H_2_O, 0.5 g L^−1^ KH_2_PO_4_, 1 g L^−1^ yeast extract (Merck Millipore, Germany) 1 mL L^−1^ vitamin solution, and 1 mL L^−1^ trace element solution and varying concentrations of glucose and NH_4_Cl as indicated. During cultivation, pH 6.0 was maintained by automatic addition of 10 M NaOH or pH was kept above 6.2 by manual addition of CaCO_3_. The stirring rate was kept constant at 1000 rpm with 2 Rushton impeller. The bioreactor was aerated with an aeration rate of 1 L min^−1^ (2 vvm) for working volume of 0.5 L or 2 L min^−1^ (1 vvm) for total volume of 2 L, while evaporation was limited by sparging the air through a water bottle. The temperature was set at 30 °C. The bioreactor was inoculated to a final OD_600_ of 0.75 with cells from an overnight culture in 50 mL screening medium containing 50 g L^−1^ glucose and 100 mM MES buffer.

### Analytical methods

When using CaCO_3_ as buffer, 1 mL of culture broth was taken for OD_600_ determination and HPLC analysis. The CaCO_3_ was dissolved with HCL prior to further measurements, basically as described by Zambanini et al. [77].

Cell densities were measured by determining the absorption at 600 nm with an Ultrospec 10 Cell Density Meter (Amersham Biosciences, Chalfont St Giles, UK).

For CDW determination of controlled high-density pulsed fed-batch fermentation of *U. maydis* MB215 ∆*cyp3* ∆P_*ria1*_::P_*etef*_ ∆*fuz7* P_*etef*_*mttA* with NaOH titration 1 mL culture broth was centrifuged at maximum speed (Heraeus Megafuge 16R, TX-400 rotor, Thermo Scientific) and the pellet was dried (Scan Speed 40 lyophilizer, Labogene ApS) for 24 h at 38 °C and weighed afterwards.

Off-gas analysis for online monitoring of CO_2_ content were performed with BCpreFerm sensors (BlueSens gas sensor GmbH).

Differential interference contrast (DIC) microscopy was performed with a Leica DM500 light microscope (Leica Microsystems). Images were recorded with a Leica ICC50 digital microscope camera (Leica Microsystems). Images were taken at 630-fold magnification. The cell morphology was analyzed by microscopy at different time points in all cultivations.

The ammonium concentration in the culture supernatant was measured by a colorimetric method according to Willis et al. [73] using salicylate and nitroprusside.

Products in the supernatants were analyzed in a DIONEX UltiMate 3000 High-Performance Liquid Chromatography System (Thermo Scientific, Germany) with an ISERA Metab AAC column 300 × 7.8 mm column (ISERA, Germany). As solvent, 5 mM H_2_SO_4_ with a flow rate of 0.6 mL min^−1^ and a temperature of 40 °C was used. Samples were filtered with Rotilabo^®^ (CA, 0.20 µm, *Ø* 15 mm) or Acrodisc^®^ (GHP 0.20 µm, Ø 13 mm) syringe filters and afterwards diluted up to 1:30 with 5 mM H_2_SO_4_. Itaconate and malate were determined with a DIONEX UltiMate 3000 Variable Wavelength Detector set to 210 nm, and glucose with a refractive index detector SHODEX RI-101 (Showa Denko Europe GmbH, Germany). Analytes were identified via retention time and UV/RI quotient compared to corresponding standards. All values are the arithmetic mean of at least three biological replicates instead of CaCO_3_ fermentations (*n* = 1). Error bars indicate the deviation from the mean for *n* = 2, if *n* > 2 error bars indicate the standard error of the mean. Statistical significance was assessed by *t*-test (two-tailed distribution, heteroscedastic, *p* ≤ 0.05).

### Plasmid cloning and strain engineering

Plasmids were assembled by Gibson assembly [22] using the NEBbuilder HiFi DNA Assembly kit (New England Biolabs, Ipswich, MA, USA). Primers were ordered as unmodified DNA oligonucleotides from Eurofins Genomics (Ebersberg, Germany). As polymerase, Q5 High-Fidelity Polymerase was used. Detailed information about utilized primers and plasmid are listed in Additional file 1: Table S3 and S4. All assembled plasmids were subcloned into *E. coli* 10β from New England Biolab and confirmed by PCR, restriction or sequencing. Standard cloning techniques for *E. coli* were performed according Sambrook et al. [64]. For transformation, preparation of protoplasts and isolation of genomic DNA of *U. maydis* protocols according to Brachmann et al. [8] were used. For deletion of *fuz7* in *U. maydis*, homologous recombination with 1000 bp flanking regions (F1, F2) including FRT sites and a hygromycin resistance cassette were used. For integration of pETEF_CbxR*_At_mttA* [32], the plasmid was linearized with SspI and integrated into the genome. For exchange of the promoter of *ria1,* CRISPR/Cas9 system was used according to Schuster et al. [65] and sgRNA has been selected online with http://www.e-crisp.org/E-CRISP/ [28]. A donor template was used to exchange the native promoter with the strong and constitutive P_*etef*_. Successful integration and deletion were verified by PCR and sequencing.

## Supplementary information

**Additional file 1. Figure S1:** Itaconate production from glycerol; Figure S2: NaOH-titrated fed-batch fermentation; Tables S1 and S2: Feeding procedures during high-density pulsed fed-batch fermentations; Table S3: Primers used in this work. Table S4: Plasmids used in this work.

## Data Availability

The datasets generated during and/or analyzed during the current study are available from the corresponding author on reasonable request.

## Abbreviations

CRISPR: clustered regularly interspaced short palindromic repeats
FLP/FRT: flippase-mediated recombination
pH: potential hydrogen
ITA: itaconic acid
GLC: glucose
HCl: hydrochloric acid
Ca-itaconate: calcium itaconate
C-source: carbon source
h: hour
HPLC: high performance liquid chromatography
CDW: cell dry weight
UV/RI: ultraviolet/refractive index
PCR: polymerase chain reaction
*A. terreus*: *Aspergillus terreus*
*E. coli*: *Escherichia coli*
*A. niger*: *Aspergillus niger*
*C. glutamicum*: *Corynebacterium glutamicum*
*U. maydis*: *Ustilago maydis*
*U. cynodontis*: *Ustilago cynodontis*
*U. vetiveriae*: *Ustilago vetiveriae*
*ria1*: regulator of itaconic acid biosynthesis
*mtt1*: mitochondrial transporter from *Ustilago maydis*
*mttA*: mitochondrial transporter from *Aspergillus terreus*
*fuz7*: dual specificity protein kinase
*cyp3*: oxidase encoding gene
P_*ria1*_: native promoter of *ria1*
P_*etef*_: strong and constitutive promoter
MES: 2-(*N*-morpholino) ethanesulfonic acid
OD: optical density
