# Supplementary material for: Integrated strain- and process design enable production of 220 g L−1 itaconic acid with Ustilago maydis
Source: Biotechnol Biofuels. 2019 Nov 6;12:263. doi: 10.1186/s13068-019-1605-6 (PMC6833137; doi:10.1186/s13068-019-1605-6)
Supplement: Supplementary file 1 — Additional file 1. Figure S1: Itaconate production from glycerol; Figure S2: NaOH-titrated fed-batch fermentation; Tables S1 and S2: Feeding procedures during high-density pulsed fed-batch fermentations; Table S3: Primers used in this work. Table S4: Plasmids used in this work. [file 13068_2019_1605_MOESM1_ESM.docx]

Additional data to:

**Integrated strain- and process design enable production of 220 g L^-1^ itaconic acid with *Ustilago maydis***

Hamed Hosseinpour Tehrani^1^, Isabel Bator^1^, Katharina Saur^1^, Svenja Meyer^1^, Ana Catarina Rodrigues Lóia, Lars M. Blank^1^ & Nick Wierckx*^1, 2^

*to whom correspondence should be addressed

^1^ iAMB – Institute of Applied Microbiology, ABBt – Aachen Biology and Biotechnology, RWTH Aachen University, Worringerweg 1, 52074 Aachen, Germany

^2^ Institute of Bio- and Geosciences IBG-1: Biotechnology, Forschungszentrum Jülich, 52425 Jülich, Germany

Address for correspondence:

Prof. Dr. Nick Wierckx

Phone: +49 246161 85247

Fax: +49 246161 2710

email: n.wierckx@fz-juelich.de

**Additional figures**

Fig. S1. Itaconate production and growth of engineered *U. maydis* strains on glycerol. Itaconate concentration (A), growth (B) and macroscopic image of a 24 well-plate (C) of cultures of *U. maydis* ∆*cyp3* P*_etef_ria1* (C_(a1-3)_), ∆*cyp3* ∆P*_ria1_*::P*_etef_* (C_(a4-6)_), ∆*cyp3* ∆P*_ria1_*::P*_etef_* *∆fuz7* (C_(b1-3)_) and ∆*cyp3* ∆P*_ria1_*::P*_etef_* *∆fuz7* P*_etef_mttA* (C_(b4-6)_) during System Duetz^®^ cultivation in screening medium with 33 g L^-1^ CaCO_3_ and 112 g L^‑1^ glycerol. Error bars indicate the standard error of the mean (n=3).


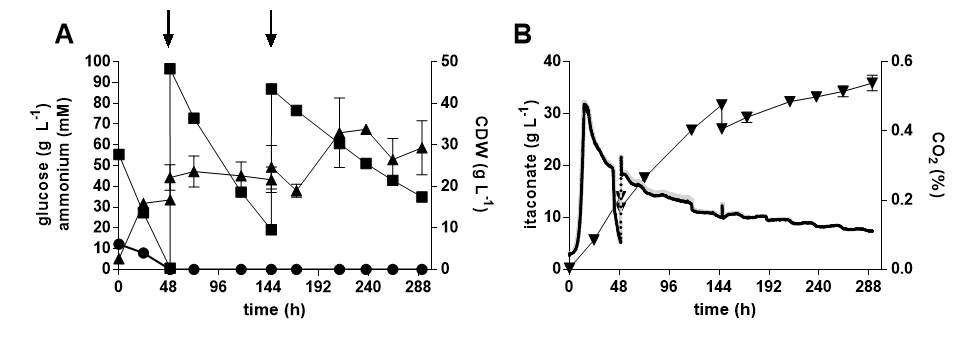


Fig. S2. Controlled high-density pulsed fed-batch fermentation of *U. maydis* MB215 ∆*cyp3* ∆P*_ria1_*::P*_etef_* ∆*fuz7* P*_etef_mttA* with NaOH titration. A: CDW (▲), glucose (■) and ammonium concentration (●) and B: Concentration of itaconate (▼) and offgas CO_2_ (continuous line) during fermentation in a bioreactor containing batch medium with glucose, 0.8 g L^-1^ NH_4_Cl at pH 6.0 titrated with NaOH. Arrows indicate addition of 100 mL of 50 % glucose. Error bars indicate the error from the mean (n=3).

**Additional tables**

Table S1. Feeding procedure during high-density pulsed fed-batch fermentation of *U. maydis* MB215 ∆*cyp3* ∆P*_ria1_*::P*_etef_* . ∆*fuz7* P*_etef_mttA*. CaCO_3_ and glucose feed protocol during fermentation in a bioreactor containing batch medium with glucose, 1.6 g L^-1^ NH_4_Cl above pH 6.0 controlled with CaCO_3_.

| **feed** | **hours (h)** | **glucose (mL)** | **CaCO_3_** |
| --- | --- | --- | --- |
| 1 | 24 | 100 |  |
| 2 | 58 | 80 |  |
| 3 | 95 | 50 |  |
| 4 | 96 |  | 50 mL (L)^a^ |
| 5 | 102 |  | 50 mL (L)^a^ |
| 6 | 120 | 50 |  |
| 7 | 151 |  | 50 mL (L)^a^ |
| 8 | 167.5 |  | 25 mL (L)^a^ |
| 9 | 173.5 | 25 |  |
| 10 | 191 | 25 |  |
| 11 | 201 | 30 | 30 mL (L)^a^ |
| 12 | 223 | 30 |  |
| 13 | 247 | 30 |  |
| 14 | 269.5 | 30 | 30 mL (L)^a^ |
| 15 | 311.5 |  | 50 mL (L)^a^ |
| 16 | 313.5 | 30 |  |
| 17 | 315.5 |  | 50 g (S)^b^ |
| 18 | 360.5 | 30 |  |
| 19 | 387.5 | 30 |  |
| ∑ (g) |  | 432 | 192.5 |

^a^ L: Liquid

^b^ S: Solid

Table S2. Feeding procedure during high-density pulsed fed-batch fermentation of *U. maydis* MB215 ∆*cyp3* ∆P*_ria1_*::P*_etef_* . ∆*fuz7* P*_etef_mttA*. CaCO_3_ and glucose feed protocol during fermentation in a bioreactor containing batch medium with glucose, 4.0 g L^-1^ NH_4_Cl above pH 6.0 controlled with CaCO_3_.

| **feed** | **hours (h)** | **glucose (mL)** | **CaCO_3_** |
| --- | --- | --- | --- |
| 1 | 45 |  | 50 mL (I)^a^ |
| 2 | 46.5 |  | 50 mL (I)^a^ |
| 3 | 68.5 | 50 |  |
| 4 | 70.5 | 60 |  |
| 5 | 77 | 100 |  |
| 6 | 116 | 60 | 50 mL (I)^a^ |
| 7 | 124.5 | 30 |  |
| 8 | 141 |  | 50 mL (I)^a^ |
| 9 | 147 | 30 | 30 mL (I)^a^ |
| 10 | 169.5 | 50 |  |
| 11 | 189 |  | 50 mL (I)^a^ |
| 12 | 191 | 100 |  |
| 13 | 193 |  | 50 g (s)^b^ |
| 14 | 238 | 100 |  |
| 15 | 265 |  | 50 g (s)^b^ |
| 16 | 286.5 | 100 |  |
| 17 | 335 |  | 50 g (s)^b^ |
| 18 | 358.5 | 100 |  |
| 19 | 383.5 | 100 | 50 g (s)^b^ |
| ∑ (g) |  | 704 | 340 |

^a^ L: Liquid

^b^ S: Solid

Table S3. Primer. The following Primer were used as PCR primers for cloning, diagnostic PCRs and sequencing procedures. Shown are the respective designations, description and sequence. Specific binding sequence are capitalized and lower case letters indicate overhangs.

|  | **Sequence (5´ 🡪 3´)** | **description** |
| --- | --- | --- |
| pJET1.2-fwd_URA_ | CGACTCACTATAGGGAGAGCGGC | for sequencing and verification of pTARGET-P*_URA_* |
| pJET1.2-fwd_URA_ | AAGAACATCGATTTTCCATGGCAG | for sequencing and verification of pTARGET-P*_URA_* |
| HT-12 | CGTTGTAGAATGGAATTTTG | fwd. primer for amplification of pTARGET-P*_ria1_* |
| HT-82 | gagcttcatgatacggatcgGTTTTAGAGCTAGAA | rev. primer for amplification of pTARGET-P*_ria1_* |
| pJET1.2-fwd_P_*_ria1_* | CGACTCACTATAGGGAGAGCGGC | for sequencing and verification of pTARGET-P*_ria1_* |
| pJET1.2-fwd *_ria1_* | AAGAACATCGATTTTCCATGGCAG | for sequencing and verification of pTARGET-P*_ria1_* |
| HT-8a | GTCGAGCTCGGTACGGGT | fwd. primer for amplification of CRISPR_P*_etef_* target |
| HT-42 | CAAAATTCCATTCTACAACG | rev. primer for amplification of CRISPR_P*_etef_* target |
| HT-9 | CCTTGCAATTCGCGCACACC | rev. primer for verification of right assembly of pCas9_P*_etef_*_1 |
| HT-10 | GCTCGGTACGGGTACTAATG | rev. primer for verification of right assembly of pCas9_P*_etef_*_1 |
| HT-43 | AAAGTGTGCCGCAGGTGAGG | for sequencing and verification of pCas9_P*_etef_*_1 |
| HT-83 | ctcgagtttttcagcaagatTTTGGTGCGATCTCGTTC | fwd. primer for amplification of F1-P*_oma_* |
| HT-84 | atccccggccATGCGCTTTGCAGGGATG | rev. primer for amplification of F1-P*_oma_* |
| HT-87 | ctcgagaagtGTATCTGGCCAGCCAGCC | fwd. primer for amplification of F2-P*_oma_* |
| HT-88 | aggagatcttctagaaagatGGTCGAGCCAGGCGCATG | rev. primer for amplification of F2-P*_oma_* |
| HT-85 | caaagcgcatGGCCGGGGATCCTGATAG | Fwd. primer for amplification of P*_oma_* from pUMa 2326 |
| HT-86 | ggccagatacACTTCTCGAGCAGGGGGATTC | rev. primer for amplification of P*_oma_*  from pUMa 2326 |
| HT-147 | ctcatccctgcaaagcgcatTGGATGATGTTGTCTGTGTATGGTATG | fwd. primer for amplification P*_etef_*  from pETEF-05080_CbxR |
| HT-148 | atggctggctggccagatacAGCTTGCATGCCTGCAGG | rev. primer for amplification P*_etef_*  from pETEF-05080_CbxR |
| HT-89 | TTTGGTGCGATCTCGTTC | fwd. primer for amplification on donor construct |
| HT-90 | GGTCGAGCCAGGCGCATG | rev. primer for amplification on donor construct |
| HT-204 | ctcgagtttttcagcaagatCCGATCGCTGTTAGGACAC | fwd. primer for amplification of F1-*fuz7* to construct pFRT^WT^-*Um*_*fuz7* |
| HT-205 | acttctggccCGTGAAACGTTGCAAAACAG | rev. primer for amplification of F1-*fuz7* to construct pFRT^WT^-*Um*_*fuz7* |
| HT-208 | acttctggccCCGACTGAGAGATTATGGTC | fwd. primer for amplification of F2-*fuz7* to construct pFRT^WT^-*Um*_*fuz7* |
| HT-209 | aggagatcttctagaaagatAATCGGAACCGTGTACCTG | rev. primer for amplification of F2-*fuz7* to construct pFRT^WT^-*Um*_*fuz7* |
| HT-206 | acgtttcacgGGCCAGAAGTTCCTATTC | fwd. primer for amplification of FRT^WT^+Hyg^R^_*fuz7* to construct pFRT^WT^-*Um*_*fuz7* |
| HT-207 | tctcagtcggGGCCAGAAGTTCCTATAC | rev. primer for amplification of FRT^WT^+Hyg^R^_*fuz7* to construct pFRT^WT^-*Um*_*fuz7* |
| HT-210 | TCGCTGTTAGGACACAACTG | fwd. primer to amplify deletion construct of *fuz7* from pFRT^WT^-*Um*_*fuz7* |
| HT-211 | CCGTGTACCTGGCTGTGTAG | rev. primer to amplify deletion construct of *fuz7* from pFRT^WT^-*Um*_*fuz7* |
| HT-4a | ACAGACGTCGCGGTGAGTTC | fwd. primer for verification of *fuz7* deletion in *U. maydis* |
| HT-212 | GGATCCCGTGGATGATGTTG | rev. primer for verification of *fuz7* deletion in  *U. maydis* |

Table S4**.** Plasmids

| plasmid | characteristics / description | assembly description | Reference |
| --- | --- | --- | --- |
| pCas9_sgRNA_0 | ori: origin of replication, bla: b-lactamase gene, ARS: autonomously replicating, cbx^R^, *U. maydis* U6 promoter, P*_otef_*: strong constitutive promoter, Tnos: nos terminator |  | (Schuster et al., 2016) |
| pTARGET-P*_URA_* | CRISPR_P_URA_ target (20 bp region upstream of the Acc65I site in pCas9_ sgRNA_0, respective sgRNA sequence, the guide RNA scaffold, the U6 teminator, a 34 nucleotide stuffer sequence and the 20 bp region downstream of Acc65I site in pCas9_ sgRNA_0) (Schuster et al., 2016). | CRISPR_P_URA_ target was synthesized by Thermo Fischer and subcloned in pJET1.2/blunt vector. | this study |
| pTARGET-P*_ria1_* | CRISPR_P*_etef_* target (20 bp region upstream of the Acc65I site in pCas9_ sgRNA_0, respective sgRNA sequence, the guide RNA scaffold, the U6 teminator, a 34 nucleotide stuffer sequence and the 20 bp region downstream of Acc65I site in pCas9_ sgRNA_0) (Schuster et al., 2016). | Self-ligation; pTARGET-P_URA_ was amplified with phosphorylated primer HT-12 and HT-82 following by a self-liagtion. | this study |
| pCas9_P*_etef_*_1 | pCas9_sgRNA_0 + sgRNA_P*_ria1_* | HIFI DNA assembly; CRISPR_P*_etef_* target was amplified from pTARGET-P*_ria1_* and pCas9_sgRNA_0 was linearized with Acc65I. Afterwards both Fragments were assembled. | this study |
| pDONOR_P*_oma_ria1* | pJET1.2/blunt with constitutive promoter P*_oma_* and 1000 bp flanking regions. F1 is in Chr04 at 37427-38426 and F2 is in Chr04 at 39761-40760 | HIFI DNA assembly; F1 and F2 was amplified from *U. maydis* genome and P*_oma_* from pUMa 2326. Afterwards all Fragments were assembled. | this study |
| pDONOR_P*_etef_ria1* | pJET1.2/blunt with constitutive promoter P*_etef_* and 1000 bp flanking regions. F1 is in Chr04 at 37427-38426 and F2 is in Chr04 at 39761-40760 | HIFI DNA assembly; Backbone was amplified from pDONOR_P*_oma_ria1*, P*_etef_* was amplified from |  |
| pETEF-05080_CbxR | UMAG_05080 (regulator of itaconate, Ria1) |  | (Geiser et al., 2016) |
| pJET1.2/blunt | Rep(pMB1); ampR; *eco*47IR; P_IACUV5_; T7 promoter |  | Thermo Fischer |
| pstorI_1rh_WT(pUMa1522) | FRT_WT; Phsp70; Thsp70; hyg^R^ |  | (Terfruchte et al., 2014) |
| pFRT^WT^-*Um*_*∆fuz7* | pJET1.2/blunt with FRT^WT^-sites, hyg^R^ and 1000 bp flanking regions up and downstream from *fuz7* | HIFI DNA assembly; F1-*fuz7* and F2-*fuz7* were amplified from *U. maydis* MB215 genome, FRT^WT^ sites including hyg^R^- cassette from pstorI_1rh_WT (FRT+Hyg^R^_*ras2)*. Afterwards all fragments were assembled with pJET1.2/blunt as backbone | this study |
| pETEF_CbxR*_At_mttA* | *cbxR, mttA controlled by constitutive promoter Petef* |  | Tehrani et al. (2019) |

**References**

Geiser, E., Przybilla, S.K., Engel, M., Kleineberg, W., Buttner, L., Sarikaya, E., Den Hartog, T., Klankermayer, J., Leitner, W., Bölker, M., Blank, L.M., Wierckx, N. 2016. Genetic and biochemical insights into the itaconate pathway of *Ustilago maydis* enable enhanced production. Metab. Eng. 38, 427-435.

Schuster, M., Schweizer, G., Reissmann, S., Kahmann, R. 2016. Genome editing in *Ustilago maydis* using the CRISPR-Cas system. Fungal Genet. Biol. 89, 3-9.

Terfruchte, M., Joehnk, B., Fajardo-Somera, R., Braus, G.H., Riquelme, M., Schipper, K., Feldbrügge, M. 2014. Establishing a versatile Golden Gate cloning system for genetic engineering in fungi. Fungal Genet. Biol. 62, 1-10.
